# The Isolation, Identification, and Whole-Genome Sequencing of a Potential Probiotic, *Clostridium butyricum* YF1, Isolated from the Intestine of the Ricefield Eel (*Monopterus albus*)

**DOI:** 10.3390/ani15040511

**Published:** 2025-02-11

**Authors:** Yubo Feng, Jing Zhang, Lan Zhou, Jiali Jin, Huamei Yue, Huan Ye, Peng Fu, Ling Huang, Rui Ruan, Chuangju Li

**Affiliations:** 1 College of Fisheries and Life Science, Shanghai Ocean University, Shanghai 201306, China; 2 Key Laboratory of Freshwater Biodiversity Conservation, Ministry of Agriculture and Rural Affairs of China, Yangtze River Fisheries Research Institute, Chinese Academy of Fishery Sciences, Wuhan 430223, China; jingz@yfi.ac.cn (J.Z.); jljin@yfi.ac.cn (J.J.); yhuam@yfi.ac.cn (H.Y.); yehuan@yfi.ac.cn (H.Y.); huangling@yfi.ac.cn (L.H.); 3 Chongqing Fishery Sciences Research Institute, Chongqing 400020, China

**Keywords:** ricefield eel, *Clostridium butyricum*, short-chain fatty acids, probiotic

## Abstract

*Clostridium butyricum*, recognized as a probiotic, is widely distributed in the intestines of various animals. In this study, the *C. butyricum* strain YF1 was isolated from the intestine of the ricefield eel (*Monopterus albus*) using an anaerobic culture method and was identified through morphological, physiological, biochemical, and 16S rRNA sequence analyses. Notably, *C. butyricum* YF1 exhibited a rapid growth rate and was found to produce ten types of short-chain fatty acids, particularly high-yield acetic acid and butyric acid. Additionally, YF1 demonstrated a high tolerance to elevated temperatures (70 °C), bile salts (0.1% to 0.5%), artificial intestinal fluid, and artificial gastric fluid, while being sensitive to most antibiotics. Further whole-genome sequencing revealed that *C. butyricum* YF1 has a total genome size of 4,314,266 bp and contains 3853 coding genes. Specifically, 82 tRNAs, 21 rRNAs, 288 repeat sequences, 13 prophages, and two gene islands were detected. Moreover, gene function analysis indicated that the highest number of genes were annotated to metabolic processes, and the butyric acid metabolism pathway was found to be complete. Meanwhile, 598 virulence genes and 186 resistance genes were predicted. In conclusion, the findings from this study contribute to probiotic development and provide innovative approaches for the sustainable and healthy cultivation of ricefield eels.

## Simple Summary

The ricefield eel (*Monopterus albus*) is an economically significant fish in China, valued for its nutritious meat. However, increased breeding density, environmental degradation, and disease outbreaks pose threats to sustainable farming of ricefield eels. While antibiotics are commonly employed to manage fish diseases, their overuse can result in drug residues and the development of resistance. Recent studies indicate that *Clostridium butyricum*, when used as a feed additive, can enhance growth performance, improve feed efficiency, increase digestive enzyme activity, inhibit pathogens, modulate immune responses, and regulate intestinal microbiota in fish. In this study, a strain of *C. butyricum* was isolated from healthy ricefield eels and identified as *C. butyricum* YF1 through morphological observation, physiological and biochemical testing, 16S rDNA sequencing, and whole genome analysis. Its biological properties, including growth characteristics, short-chain fatty acid metabolites, tolerance, and drug susceptibility, were further investigated. The findings of this study provide a theoretical foundation for the future application and research of *C. butyricum* YF1.

## 1. Introduction

*Clostridium butyricum* is a rod shape, strictly anaerobic, Gram-positive bacterium that forms spores [1]. It is commonly found in healthy animals (in their gastrointestinal tracts or feces), as well as in soil, water, and fermentation products [2]. *C. butyricum* produces a variety of metabolites, including short-chain fatty acids (SCFAs), biofuel compounds, and precursors of biomaterials (such as hydrogen, butanol, and 1,3-propanediol) during the fermentation of carbohydrates and sugars [3]. In recent years, several specific strains of *C. butyricum* have been demonstrated to be effective probiotics and are widely used in aquaculture. Dietary supplementation with *C. butyricum* improves growth performance, increases feed efficiency, enhances digestive enzyme activity, inhibits pathogen invasion, modulates immune responses, improves disease resistance, regulates intestinal microorganisms, and changes the intestinal structure in triploid rainbow trout (*Oncorhynchus mykiss*) [4], common carp (*Cyprinus carpio*) [5,6], largemouth bass (*Micropterus salmoides*) [7,8,9], tilapia (*Oreochromis niloticus*) [10,11], yellow catfish (*Pelteobagrus fulvidraco*) [12,13], pacific white shrimp (*Litopenaeus vannamei*) [14,15], turbot (*Scophthalmus maximus*) [16], large yellow croaker (*Larimichthys crocea*) [17], hybrid groupers (*Epinephelus lanceolatus*♂ × *E. fuscoguttatus*♀) [18,19], and freshwater prawns (*Macrobrachium rosenbergii*) [20]. These findings highlight the potential of *C. butyricum* in aquatic animals; however, most bacterial strains originate from sources other than aquatic animals.

As reported, probiotic strains from different sources exhibit varying properties [21,22,23]. Biofilm formation enables probiotic bacteria to colonize the gut, remain active, and compete with the gastrointestinal pathogens for surface colonization. Boricha et al. (2019) found that the *Lactobacillus plantarum* strain V3F shows higher biofilm formation in the presence of sodium taurocholate compared to the *Lactobacillus pentosus* and *Lactobacillus fermentum* strains [24]. Recently, three strains of the potential probiotic *C. butyricum* were isolated from the guts of yellow catfish (*Pelteobagrus fulvidraco*) [13], crustacean mud crab (*Scylla paramamosain*) [25], and pacific white shrimp (*Litopenaeus vannamei*) [26]. However, no *C. butyricum* has yet been isolated from ricefield eels (*Monopterus albus*). The ricefield eel is an important aquaculture species in China and is classified as a protogynous hermaphroditic Synbranchiform teleost. According to the China Fishery Statistical Yearbook, the total production of ricefield eels reached 355,203 tons in 2023.

In this study, a strain designated YF1 was isolated from the intestine of ricefield eels using an anaerobic isolation technique. It was subsequently identified through morphological, physiological, biochemical, and 16S rRNA sequence analyses. The strain was further characterized by examining its growth curves and production of short-chain fatty acids (SCFAs). Meanwhile, its tolerance to high temperatures, bile salts, artificial intestinal fluid, and artificial gastric fluid was assessed, along with its sensitivity to antibiotics. Additionally, the whole genome of YF1 was sequenced. Overall, this study examined the in vitro probiotic potential of the isolated strain, establishing a scientific foundation for future research and applications involving this strain.

## 2. Materials and Methods

### 2.1. Isolation and Identification of C. butyricum YF1

The ricefield eels used in this study were obtained from the wild in Wuhan. Ricefield eels were first anesthetized by a 0.05% solution of 3-aminobenzoic acid ethyl ester methanesulfonate-222 (MS-222) (Sigma-Aldrich, St. Louis, MO, USA) before dissection. Sterile dissection of the ricefield eels was performed on ice; the intestines were aseptically collected, and the gut contents from the posterior intestine were gently scraped and diluted with 0.1 M sodium phosphate buffer (PBS) (pH 6.8; Hopebio, Qingdao, China). The diluent was placed in a water bath at 70 °C for 10 min. The samples were then incubated anaerobically in reinforced clostridial medium (RCM; Hopebio, Qingdao, China) at a concentration of 5% at 37 °C for 48 h. Subsequently, the culture was serially diluted to concentrations of 10^−4^, 10^−5^, and 10^−6^. A volume of 100 µL of the diluted bacterial solution was applied to Trypticase Sulfite Neomycin (TSN) agar medium (Hopebio, Qingdao, China) and incubated anaerobically at 37 °C for 24 h. The morphological characteristics of the colonies were observed, followed by Gram staining of suspicious colonies. Eligible colonies were selected and further purified on TSN agar medium. Bacterial morphology was examined under a light microscope (DM3000; Leica, Wetzlar, Germany) and a HITACHI-SU8100 scanning electron microscope (Hitachi, Tokyo, Japan) by Wuhan Servicebio Technology Co., Ltd. (Wuhan, China). The physiological and biochemical properties of the strain were determined by consulting Bergey’s Bacteria Identification Manual and the Manual of Identification of Common Bacterial Systems [27,28].

Bacterial genomic DNA was extracted and purified as instructed in the manuals of the kit (TIANGEN), and polymerase chain reaction (PCR) amplification was performed with the use of 16 S rDNA universal primers (27 F: 5′-AGAGTTTGATCCTGGCTCAG-3′; 1492 R: 5′-GGCTACCTTGTTACGACTT-3′). The reaction system was 25 µL, including 1 µL DNA template, 0.4 µL each of the upstream and downstream primers, 12.5 µL of 2 × Taq Master Mix, and 10.7 µL ddH_2_O. The reaction conditions were as follows: 5 min at 94 °C; 35 cycles of 30 s at 94 °C, 45 s at 55 °C, and 90 s at 72 °C; and a final 7 min incubation at 72 °C. The 16 S rDNA sequencing result was further analyzed with the Basic Local Alignment Search Tool (BLAST) from the National Center for Biotechnology Information (NCBI) database. A BLASTN alignment was performed to determine the species taxonomic information of the isolate. The 16 S rDNA sequence of the *C. butyricum* strain YF1 was uploaded to the NCBI database (GenBank: PQ524489).

### 2.2. Biological Characterization of C. butyricum YF1

#### 2.2.1. OD_600_, pH, and SCFA Content of YF1 at Different Incubation Times

Activated *C. butyricum* strains were anaerobically incubated in liquid growth medium (yeast powder 30 g/L, glucose 10 g/L, sodium chloride 2 g/L, magnesium sulfate heptahydrate 0.25 g/L, dimethyl hydrogen phosphate 2 g/L, and L-cysteine hydrochloride 0.5 g/L at pH 7.0 ± 0.1) at 37 °C. The medium was refreshed every 4 h. The optical density (OD) value at 600 nm was recorded, and the potential of hydrogen (pH) was measured. Meanwhile, the incubation broth was centrifuged at 4000× *g* for 10 min, and the supernatants were collected separately for the determination of the SCFA content by Wuhan MetWare Biotechnology Co., Ltd. (Wuhan, China). Three replicate samples were detected for each group.

#### 2.2.2. Tolerance Test

*C. butyricum* YF1 was inoculated into RCM and incubated anaerobically at 37 °C with a shaking speed of 220 rpm/min for a duration of 72 to 96 h. Cultured spores were centrifuged at 4000× *g* for 5 min to obtain a precipitate, which was then washed with PBS and resuspended. A total of 0.5 mL of the solutions were placed in water baths at various temperatures: 50, 60, 70, 80, 90, and 100 °C, for 10 min respectively. After treatment, the solution was applied to the Sulfite Iron Agar medium (Hopebio, Qingdao, China), and the number of colonies on the plate was counted after 24 h of anaerobic incubation. Three replicates were established for each treatment. The survival rate was calculated as: survival rate (%) = (number of viable colonies in temperature treatments/number of viable bacteria before treatment) × 100%.

*C. butyricum* YF1 was inoculated into RCM and incubated anaerobically at 37 °C with a shaking speed of 220 rpm/min for a duration of 72 to 96 h. Cultured spores were centrifuged at 4000× *g* for 5 min to obtain a precipitate, which was subsequently washed with PBS and resuspended. A volume of 0.2 mL of bacterial suspension was added to 2 mL of pig bile salt (Solarbio, Beijing, China) solutions at concentrations of 0.1%, 0.3%, and 0.5%. The samples were cultured anaerobically at 37 °C for 3 h to evaluate the number of viable bacteria. Three replicates were established for each treatment. The survival rate (%) was calculated as the ratio of live bacteria after bile salt treatment to the number of live bacteria in the untreated group.

Furthermore, the tolerance of gastric and intestinal fluids was evaluated. *C. butyricum* YF1 was inoculated into RCM and incubated anaerobically at 37 °C with a shaking speed of 220 rpm/min for a duration of 72 to 96 h. Cultured spores were centrifuged at 4000× *g* for 5 min to obtain a precipitate, which was then washed with PBS and resuspended. A 0.2 mL aliquot of the bacterial suspension was added to 2 mL of either artificial gastric juice or artificial intestinal juice. Samples were collected after 0, 1, 2, 3, and 4 h of anaerobic culture at 37 °C to assess the number of viable bacteria. Three replicates were established for each treatment. The survival rate (%) was calculated as the ratio of the number of viable bacteria at different time points to the number of viable bacteria at 0 h.

Artificial gastric fluid was prepared as follows: 16.4 mL of 1 M hydrochloric acid (Nanjing Chemical Reagent Co., Ltd., Nanjing, China) was added to approximately 800 mL of water, followed by the addition of 10 g of pepsin (Solarbio, Beijing, China). The mixture was stirred thoroughly, and the volume was adjusted to 1000 mL with deionized water, resulting in a pH of 1.5 ± 0.1. For the preparation of artificial intestinal fluid, 6.8 g of potassium dihydrogen phosphate (Sinopharm Chemical Reagent Co., Ltd., Shanghai, China) was dissolved in 500 mL of deionized water. A 0.4% sodium hydroxide (Sinopharm, Chemical Reagent Co., Ltd., Shanghai, China) solution was used to adjust the pH to 6.8. In a separate container, 10 g of trypsin (Solarbio, Beijing, China) was dissolved in deionized water. After thoroughly mixing the two solutions, the mixture was diluted to a final volume of 1000 mL with deionized water, ensuring the pH remains at 6.8 ± 0.1.

#### 2.2.3. Antimicrobial Susceptibility Test

The Kirby–Bauer disk diffusion method [29] was used to assess the sensitivity of isolates to thirty antibiotics. The strain was incubated anaerobically in liquid RCM at 37 °C for 24 h. A culture suspension (100 µL, 1 × 10^5^ CFU/mL) was inoculated onto Sulfite Iron Agar, with antimicrobial tablets (Hangzhou Microbial Reagent Co., Ltd., Hangzhou, China) placed at the center. The inoculation was cultured anaerobically at 37 °C for 24 h. Three replicates were performed for each treatment. Inhibition zone sizes were measured with a vernier caliper and evaluated according to the Determination of Antimicrobial Resistance in Animals and Their Products (SN/T 1944-2016) for drug sensitivity.

### 2.3. Whole Genome Sequencing and Analysis

#### 2.3.1. Bacterial Strain and DNA Extraction

A single colony of YF1 was cultured in RCM liquid medium. High-quality genomic DNA from the *C. butyricum* strain YF1 was extracted using an improved CTAB method. DNA concentration and purity were assessed with a NanoDrop 2000 spectrophotometer (Thermo Fisher Scientific, Waltham, MA, USA), and quantification was performed using a Qubit 3.0 (Life Technologies, Carlsbad, CA, USA). DNA integrity was evaluated via 0.8% agarose gel electrophoresis.

#### 2.3.2. Library Construction

After the DNA sample passed the quality tests, the genomic DNA (gDNA) was fragmented to 10 kb using a g-Tube (Covaris) and purified with 0.45× magnetic beads. A total of 2.5 µg of the purified gDNA was used to construct a sequencing library. Following the application of an exonuclease to remove the 3′ end overhang, any damage was repaired and the ends were prepared before attaching a barcode using the SMRT dumbbell-type connector. To obtain a sequencing library, the sample was filtered again using 0.45× PB magnetic beads after performing a nucleic acid exonuclease process to eliminate disconnected fragments. Finally, the quality of the library was assessed and quantified using a Qubit 3.0. Additionally, the size of the library was verified with an Agilent 2100 (Agilent, Santa Clara, CA, USA) to ensure its quality.

#### 2.3.3. Sequencing and Assembly

After passing the library inspection, PacBio Sequel II was used to sequence the library according to the target offline data volume, and then SMRT LINK 10.1.0 software was used to process the data. PacBio readings using were reassembled using the microbial assembly (smrtlink10), HGAP4 [30], and Canu (v.1.6) [31] softwares. In addition, the depth of genome coverage was analyzed through pbalign (BLASR, v0.4.1) [32]. The assembled genome sequence of *C. butyricum* YF1 has been stored in NCBI (BioProject accession number: PRJNA1178835). A circular map of the genome of YF1 was obtained using Circos (v0.64) [33].

#### 2.3.4. Genome Component Analysis

Coding gene prediction for the newly sequenced genomes was conducted using Glimmer software (version 3.02) [34], including prediction of transfer RNA (tRNA) by tRNAscan-SE software (Version 1.3.1) [35] and prediction of ribosome RNA (rRNA) using rRNAmmer software (Version 1.2) [36]. After annotation using the Rfam (Version 12.0) database, the final small nuclear RNAs (sRNA) were determined using the cmsearch program (Version 1.1rc4) [37]. Dispersed repeat sequence prediction by RepeatMasker (Version open-4.0.5) software [38], TRF (Tandem Repeats Finder, Version 4.07b) searches for tandem repeats in DNA sequences [39], MISA searches for simple sequence repeats were conducted. Clustered Regularly Interspaced Short Palindromic Repeat Sequences (CRISPR) prediction of sample genomes using Mining CRISPRs in Environmental Datasets (MinCED) was performed. IslandPath-DIOMB software (Version 0.2) [40] was used to predict gene islands. Prediction of prophages on the sample genome by phiSpy software (Version 2.3) was conducted [41].

#### 2.3.5. Gene Function Analysis

We conducted functional annotation of genes encoding proteins identified by Glimmer, primarily using protein alignment with various databases. Predicted genes were compared against the Non-Redundant Protein Database (NR), Swiss-Prot, and the Cluster of Orthologous Groups (COG) database via BLAST (blastp, evalue ≤ 1 × 10^−5^), selecting items with the highest scores. Gene Ontology (GO) annotation was based on NR results and custom scripts, while KEGG annotation was performed using KOBAS. We also utilized specialized databases, including the Carbohydrate-Active Enzymes Database (CAZy), Comprehensive Antibiotic Research Database (CARD), Virulence Factors of Pathogenic Bacteria (VFDB), and Pathogen Host Interactions Database (PHI), for protein annotation.

### 2.4. Statistical Analysis

MEGA7.0 was used for multiple sequence alignment and phylogenetic tree construction via the neighbor-joining method with 1000 bootstrap replicates. The data were analyzed by ANOVA using the IBM SPSS Statistics Subscription Trial Software Package 30.0. The Duncan method was used for multiple comparisons between groups, and *p* < 0.05 was considered statistically significant.

## 3. Results

### 3.1. Isolation and Identification of C. butyricum YF1

**Figure 1:**
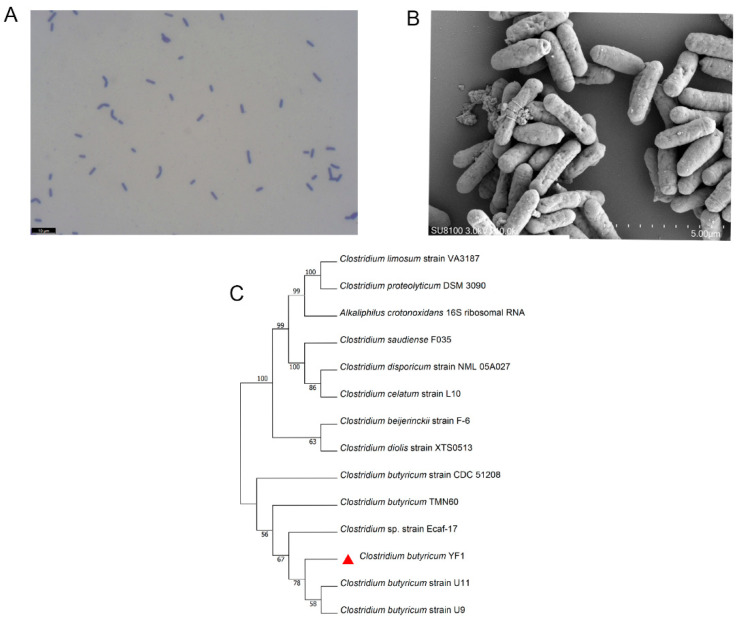
Identification of *C. butyricum* YF1. (**A**) A microscopic view of the bacterium stained using the Gram staining method; (**B**) scanning electron micrograph; and (**C**) phylogenetic tree of *C. butyricum* YF1 based on the 16S rDNA gene. Scale bars are as follows: 10 μm (**A**) and 5 μm (**B**).

An isolate was screened in RCM and TSN media. The colonies appeared creamy white and round with convex edges. Microscopic examination revealed a Gram-positive bacillus with bluntly rounded ends and a slightly expanded middle, appearing rod-shaped or slightly curved, either singly or in pairs (Figure 1A,B). The results of physiological and biochemical identification are shown Table 1. The strain tested negative for peptone water, citrate, hydrogen sulfide, and urea, while it tested positive for the other items. The strain was preliminarily identified as *C. butyricum* based on its morphological characteristics and physiological and biochemical tests. Furthermore, the amplified 16S rDNA fragment was queried against the NCBI database using a BLASTN search. The strain showed high homology with *C. butyricum* strains U11 (PQ462177.1) and U9 (PQ462176.1) (Figure 1C), confirming its identification as *C. butyricum*, and was named strain YF1 (GenBank: PQ524489).

### 3.2. Biological Characterization of C. butyricum YF1

#### 3.2.1. Growth Characteristics and Metabolites of YF1

**Figure 2:**
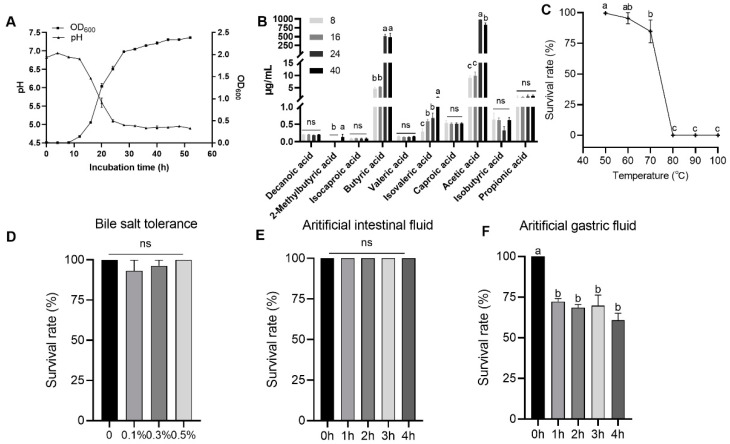
In vitro characterization of *C. butyricum* YF1. (**A**) OD_600_ and pH of YF1 at different incubation times. (**B**) YF1 yields ten short-chain fatty acids in in vitro culture. (**C**–**F**) Tolerance of YF1 to temperature, bile salt, artificial intestinal fluid, and artificial gastric fluid, respectively. Data are expressed as mean ± SEM (n = 3). Different letters indicate significant differences (*p* < 0.05). *p*-values were determined by one-way ANOVA.

As shown in Figure 2A, the growth curve of *C. butyricum* YF1 went through adjustment, logarithmic, and stabilization phases. The growth retardation period of YF1 is from 0 to 8 h; at 8 h it enters the logarithmic growth period; after 28 h it enters the growth stabilization period and the final OD_600_ value was stable at around 2.30. During the rapid reproduction period of bacteria, a large amount of acid and gas were produced, and the pH value of the supernatant of the culture medium gradually decreased and stabilized at around 4.89.

In addition, the supernatant from in vitro culture contained ten short-chain fatty acids (SCFAs). Acetic acid exhibited the highest yield (984.92 ± 81.59 mg/L), followed by butyric acid (515.42 ± 55.07 mg/L), after 24 h of culture. As the culture time increased (8, 16, 24, and 40 h), the concentrations of acetic acid, butyric acid, isovaleric acid, and 2-methylbutyric acid in the supernatant rose significantly. Acetic acid concentrations increased by 110.38 times in the 24-h group and 93.68 times in the 40-h group compared to the 8-h group. Butyric acid concentrations rose by 111.49 times in the 24-h group and 103.69 times in the 40-h group. Isovaleric acid concentrations increased by 2.01, 2.32, and 4.13 times in the 16-h, 24-h, and 40-h groups, respectively. However, 2-methylbutyric acid was detected only in the 40-h group (Figure 2B).

#### 3.2.2. Tolerance Test

The results of the temperature resistance test for YF1 are shown in Figure 2C. When the culturing temperature was below 70 °C, the activity of the spore was above 84.7%. However, the survival rate dropped sharply to 0% after the culture was exposed to 80 °C for 10 min. This indicates that 70 °C is the highest temperature at which YF1 can maintain high activity. The tolerance of *C. butyricum* YF1 to bile salts is illustrated in Figure 2D. The survival rate of YF1 spores remained above 90% after 3 h of treatment with bile salts at concentrations of 0.1%, 0.3%, and 0.5%. This indicates that the growth of YF1 was minimally affected by bile salts at concentrations up to 0.5%. The tolerance of YF1 to artificial intestinal fluid and artificial gastric fluid is depicted in Figure 2E,F, respectively. The results demonstrate that artificial intestinal fluid had no significant impact on the survival rate of YF1. However, when compared to the control group, the survival rate of YF1 spores was significantly reduced to 72%, 68%, 69%, and 60% after treatment with artificial gastric fluid for 1, 2, 3, and 4 h, respectively.

#### 3.2.3. Antimicrobial Susceptibility Test

As shown in Table 2, *C. butyricum* YF1 exhibited sensitivity to penicillin, ampicillin, carbenicillin, peracillin, cephalexin, cefazolin, cefradine, cefuroxime, ceftazidime, cefoperazone, tetracycline, doxycycline, minocycline, erythromycin, ofloxacin, ciprofloxacin, vancomycin, chloramphenicol, furazolidone, and clindamycin. The strain demonstrated moderate susceptibility to oxacillin, amikacin, kanamycin, and medemycin, while exhibiting strong resistance to ceftazidime, gentamicin, neomycin, polymyxin B, and cotrimoxazole.

### 3.3. Whole Genome Analysis of C. butyricum YF1

#### 3.3.1. The Genome Overview Component Characteristics of YF1

**Figure 3:**
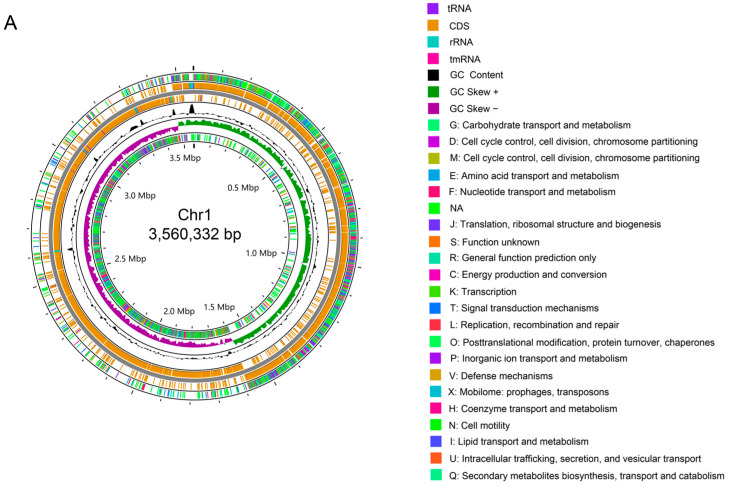

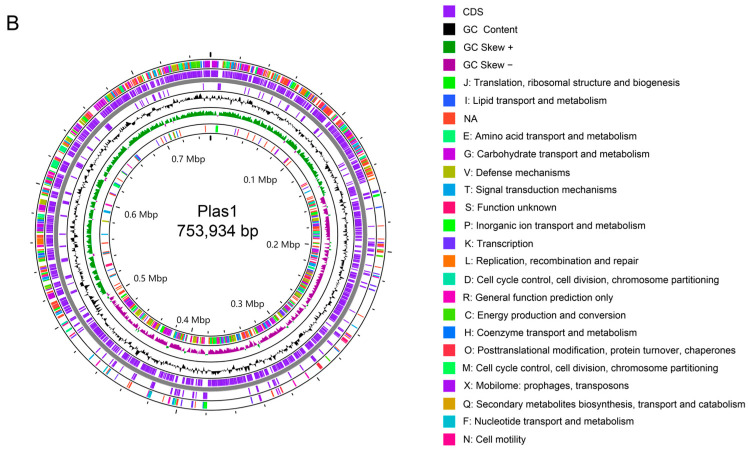
Whole-genome visualization map of *C. butyricum* YF1. (**A**) The circular chromosome genome-wide map. (**B**) The circular plasmid genome-wide map. The outermost circle represents the Cluster of Orthologous Groups (COG) functional annotation classification of genes on the positive strand. Moving from the outer edge to the inner circle, the map indicates the following: coding genes (positive strand), coding genes (negative strand), GC content, GC skew (positive), GC skew (negative), and the COG functional annotation classification of genes on the negative strand.

The total size of the genome of *C. butyricum* YF1 was identified as 4,314,266 bp (BioProject accession number: PRJNA1178835) using PacBio sequencing. This includes a circular chromosome measuring 3,560,332 bp (28.71% GC) and one circular plasmid of 753,934 bp (28.20% GC) (Figure 3A,B). A total of 3173 predicted coding genes were found in the circular chromosome, while 680 were identified in the circular plasmid. Additionally, 103 RNAs (82 tRNAs, 21 rRNAs) were obtained. A total of 288 repeat sequences were detected, consisting of 257 tandem repeats identified by Tandem Repeats Finder (TRF) and 31 Simple Sequence Repeats (SSR). The 3-base repeat (P3) and 6-base repeat (P6) predominated among the SSRs. Thirteen prophages were predicted, with a total length of 536,752 bp and an average length of 41,288 bp, including 573 genes. Gene island (GI) predictions yielded two GIs, one measuring 34,449 bp and containing 18 genes, and the other measuring 12,473 bp and containing 9 genes.

#### 3.3.2. The Genomic Functional Annotation of YF1

**Figure 4:**
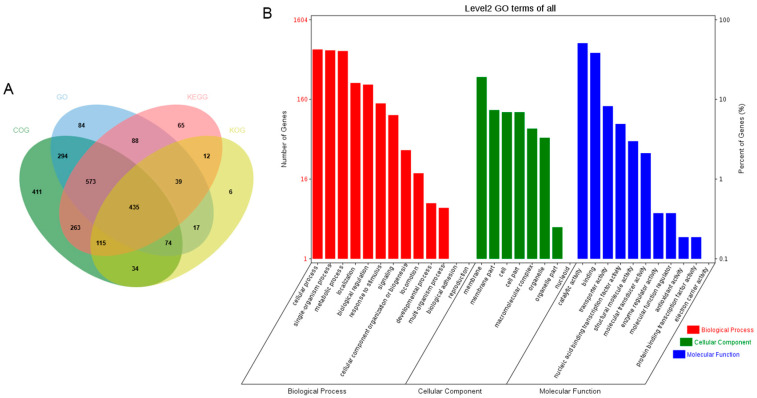
Gene function analysis of *C. butyricum* YF1. (**A**) The number of the predicted genes on the circular chromosome were annotated by COG, GO, KEGG and KOG. (**B**) GO items classification of the YF1 genome.

The coding sequences of the predicted genes on the circular chromosome were annotated by different functional annotation databases, including Clusters of Orthologous Groups (COG) (2200), Gene Ontology (GO) (1605), Kyoto Encyclopedia of Genes and Genomes (KEGG) (1591), Eukaryotic Orthologous Groups of proteins (KOG) (733), Non-redundant Protein (NR) (3143), Pfam (2635), Swiss-Prot (1976), and TrEMBL (3128) (Figure 4A).

**Figure 5:**
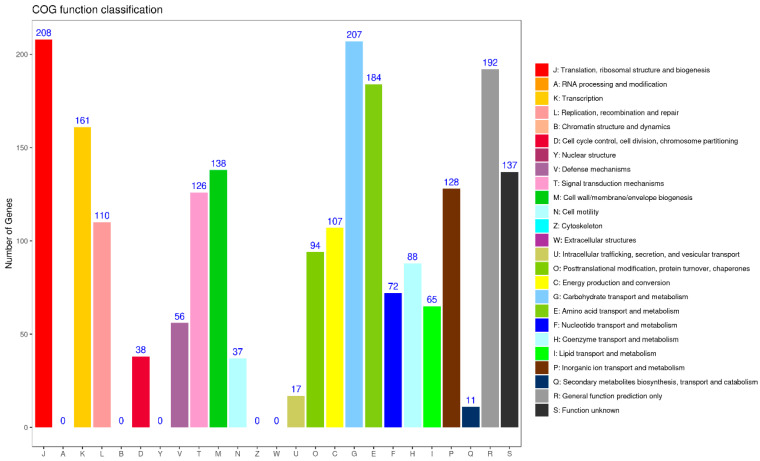
COG functional classification of the YF1 genome.

**Figure 6:**
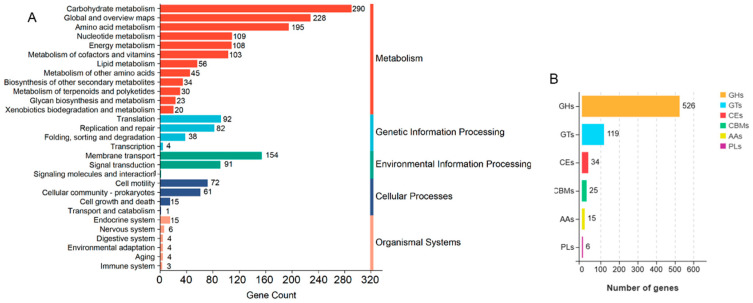
KEGG metabolic pathway classification (**A**) and CAZy annotation (**B**) of the YF1 genome. GHs: glycoside hydrolases; GTs: glycosyl transferases; CEs: carbohydrate esterases; CBMs: carbohydrate-binding modules; AAs: auxiliary activities; PLs: polysaccharide lyases.

In the GO annotation, the most prominent categories associated with the circular chromosome were cellular process; membrane and catalytic activity, as classified under the biological process; cellular component; and molecular function (Figure 4B). The results of the clusters from the COG functional annotation are presented in Figure 5. The most abundant terms were translation, ribosomal structure, and biogenesis (208), followed by carbohydrate transport and metabolism (207), and general function prediction only (192). In the KEGG annotation, the predominant metabolic pathways were carbohydrate metabolism (290), followed by global and overview maps (228), and amino acid metabolism (195) (Figure 6A). Additionally, the genome sequence of YF1 was analyzed using the Carbohydrate-Active Enzyme (CAZy) database. The results indicated that the highest number of genes on the circular chromosome were annotated as glycoside hydrolases (GHs) (526) (Figure 6B).

#### 3.3.3. Analysis of the Butyric Acid Metabolism Pathway in YF1

**Figure 7:**
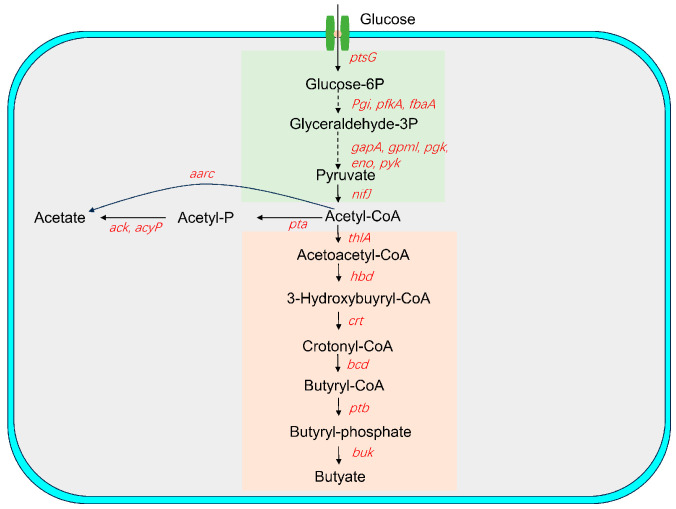
The butyric acid metabolism pathway of *C. butyricum* [42,43,44]. Italicized gene names in red represent key enzymes involved in the metabolic pathways present in the *C. butyricum* YF1 genome. Abbreviations: *ptsG*: phosphotransferase; *pgi*: glucose-6-phosphate isomerase; *pfkA*: 6-phosphofructokinase; *fbaA*: fructose-bisphosphate aldolase; *gapA*: glyceraldehyde-3-phosphate dehydrogenase; *gpml*: probable phosphoglycerate mutase; *pgk*: pyruvate kinase; *eno*: enolase; *pyk*: pyruvate kinase; *nifJ*: ferredoxin oxidoreductase; *thlA*: acetyl-CoA C-acetyltransferase; *hbd*: 3-hydroxybutyryl-CoA dehydrogenase; *crt*: crotonase; *bcd*: acyl-CoA dehydrogenase; *ptb*: phosphate butyryltransferase; *buk*: butyrate kinase; *pta*: phosphate acyltransferase; *ack*: acetate kinase; *acyP*: acylphosphatase; and *aarc*: acetate CoA-transferase;.

*C. butyricum* YF1 can produce ten types of short-chain fatty acids, with particularly high yields of acetic acid and butyric acid. In light of this, we analyzed the key enzymes involved in the metabolic pathways that generate butyric acid and acetic acid. Key enzyme genes in the glycolytic pathway, along with those involved in the synthesis of acetic and butyric acids, have been identified in the genome of the *C. butyricum* YF1 (Figure 7).

#### 3.3.4. Profiling of Resistance Genes and Virulence Factors of YF1

**Figure 8:**
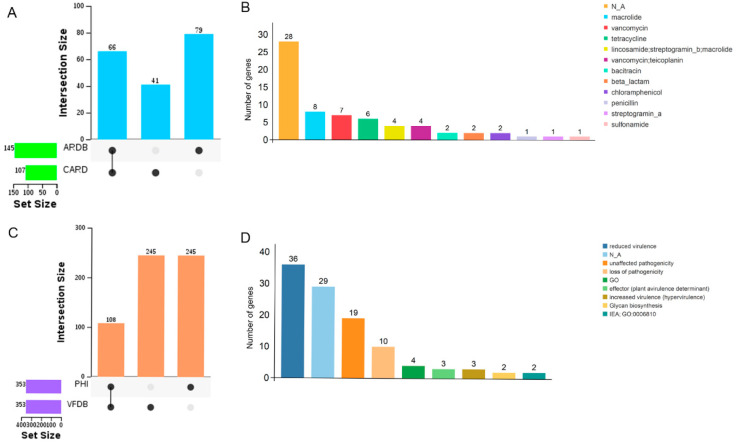
The profiling of resistance and virulence genes of YF1. (**A**) The number of resistance genes on the circular chromosome as annotated by the ARDB and CARD. (**B**) The antibiotic resistance annotation of the 66 genes. (**C**) The number of pathogenicity genes on the circular chromosome as annotated by the PHI and VFDB. (**D**) The type of phenotypic mutation of the 108 pathogenic genes.

The predicted antibiotic resistance genes on the circular chromosome, based on the Comprehensive Antibiotic Resistance Database (CARD) and Antibiotic Resistance Genes Database (ARDB), were 107 and 145 genes, respectively (Figure 8A). Among them, 66 genes are found in both CARD and ARDB, which confer resistance to macrolides, vancomycin, tetracycline, teicoplanin, lincosamide, streptogramin, chloramphenicol, β-lactams, bacitracin, sulfonamides, and penicillin (Figure 8B). Additionally, 22 genes located on the circular plasmid were co-predicted in the CARD and ARDB, which confer resistance to lincosamide, streptogramin, macrolide, vancomycin, teicoplanin, vancomycin, bacitracin, chloramphenicol, antimicrobials, neomycin, and tetracenomycin.

To analyze the pathogenicity of *C. butyricum* YF1, the virulence genes on its circular chromosome were predicted based on the Pathogen Host Interactions (PHI) and Virulence Factors of Pathogenic Bacteria (VFDB) databases. A total of 353 virulence genes were predicted from each database (Figure 8C). Among them, 108 virulence-related genes co-predicted in PHI and VFDB, which phenotypic mutation type include reduced virulence, unaffected pathogenicity, loss of pathogenicity, increased virulence (hypervirulence), effector (plant avirulence determinant), and glycan biosynthesis (Figure 8D). Additionally, 21 virulence genes located on the circular plasmid were co-predicted in PHI and VFDB, which exhibit phenotypic mutations including unaffected pathogenicity, reduced virulence, loss of pathogenicity, and increased virulence (hypervirulence).

## 4. Discussion

*C. butyricum*, a probiotic, is currently more often studied in aquatic animals in the form of a dietary supplementation. However, most strains originated from other sources rather than the aquatic animals, which may limit the beneficial effects. In the present study, a strain of *C. butyricum* was isolated from the intestine of *M. albus* and identified through morphological observation, physiological and biochemical tests, as well as 16S rDNA sequencing. The strain in our study was named YF1, which has the typical morphological, physiological, and biochemical characteristics of *C. butyricum*, which is consistent with the results of previous studies [26,45]. The result of the phylogenetic tree of 16S rDNA revealed that YF1 was highly similar to *C. butyricum* strains U11 and U9.

To investigate the biological characteristics of YF1, we first measured its growth curve. The *C. butyricum* YF1 entered the logarithmic phase at 8 h and reached the plateau phase at 28 h, indicating that *C. butyricum* YF1 has a rapid growth and reproduction rate. Zhang et al. (2024) found that the *C. butyricum* B3 isolated from the intestine of *Pelteobagrus fulvidraco* enters the logarithmic growth phase after 4 h and the stable phase at 26 h [13]. Wang et al. (2023) reported that the *C. butyricum* LV1 from *Litopenaeus vannamei* enters the logarithmic growth phase after 4 h and the stable phase at 12 h [13]. *C. butyricum* can ferment carbohydrates to produce short-chain fatty acids (SCFAs), especially butyric acid, which are used for the regeneration and repair of the intestinal epithelium and regulation of intestinal health microecosystems [46,47,48]. In this investigation, the results showed that *C. butyricum* YF1 in the in vitro incubation could increase the content of SCFAs, especially high-yield acetic acid and butyric acid. The result is consistent with the previous finding in *C. butyricum* strain B3 isolated from yellow catfish (*Pelteobagrus fulvidraco*) [13], which revealed the highest acetic acid and butyric acid production after 24 h. Notably, in this study, YF1 produces more acetic acid than butyric acid, likely due to the ATP generated from the acetic acid production pathway, which supports its growth requirements [49]. Moreover, the concentration of butyric acid increased to 515.42 ± 55.07 mg/L at 24 h, where it decreased to 479.38 ± 121.12 mg/L after 40 h of culture. This may be related to YF1 reaching a plateau phase after 28 h. This is similar with the *C. butyricum* strains B3 [13] and LV1 [26], for which the production of butyric acid no longer increased and even decreased after 24 h. Meanwhile, the concentrations of isovaleric acid and 2-methylbutyric acid in the supernatant significantly increased with longer culture times of YF1. The rise in these metabolites might also contribute to enhancing intestinal immune function in the ricefield eel [50].

Probiotic preparations are used as feed additives and are subjected to high temperatures for short durations during the feed pelleting process [51]. Furthermore, probiotics need to tolerate the environment of the digestive tract in animals because they must pass through the digestive tract and then enter the intestine to perform their probiotic functions [52,53]. Therefore, the resistance of *C. butyricum* YF1 to high temperatures, bile salts, artificial gastrointestinal fluids, and its antibacterial activity were assessed in the present study. The thermal tolerance results indicated that 80 °C is the growth-limiting temperature for YF1, suggesting that while YF1 exhibits some resistance to heat, its ability to withstand high temperatures is limited. This finding is comparable to that of *C. butyricum* LY33 [54], which can maintain high activity only up to 75 °C. In addition, the survival rate of YF1 exceeded 90% after 3 h of treatment with bile salts (0.1 to 0.5%) and artificial intestinal, while it was nearly 70% after 3 h treatment with artificial gastric fluid. These results indicate that *C. butyricum* YF1 has probiotic potential and can colonize and grow in the intestinal environment, which is consistent with the findings of existing studies [26,54]. Moreover, we found that *C. butyricum* YF1 exhibits high sensitivity to most antibiotics, indicating that this bacterium has a low risk of horizontally transferring of antibiotic resistance genes to other bacteria [55,56]. This finding is consistent with reports regarding *C. butyricum* strains B3 [13], G13 [25], and LY33 [54].

Whole-genome sequencing technology allows for a detailed genetic analysis of microorganisms, which is crucial for assessing probiotic safety and efficacy [57,58]. Yang et al. (2024) analyzed 14 assembled strains and 139 strains from NCBI, finding *C. butyricum* genome sizes between 3.74 MB and 5.29 MB, with an average GC content of 28.6%. They predicted and annotated genes from 153 *C. butyricum* strain genomes, with gene counts ranging from 3426 (EBI) to 5873 (INCQS635) and a median of 4256. Corresponding predicted proteins ranged from 3312 to 5736 [44]. In this study, *C. butyricum* isolates were subjected to whole-genome sequencing using PacBio third-generation high-throughput sequencing technology. The *C. butyricum* YF1 genome is 4,314,266 bp, comprising a circular chromosome (28.71% GC) and one circular plasmid (28.20% GC), with 3853 predicted coding genes. The chromosome of YF1 has 82 tRNAs and 21 rRNAs, comparable to *C. butyricum* DKU_butyricum 4-1 [59] and DKU-11 [60], which have 88 tRNAs and 36 rRNAs, and 88 tRNAs and 33 rRNAs, respectively. Additionally, the YF1 genome features two gene islands, indicating plasticity and recombination, and contains thirteen prophages that may influence its toxicity or antibiotic resistance. Likewise, Wang et al. (2024) found that the predicted number of gene islands and prophages in *C. butyricum* LV1 was 18 and 10, respectively [26]. The CRISPR system is vital for defense against invasive genetic elements such as phages and plasmids [61]. Yang et al. (2024) found that 60 strains of *C. butyricum* contained at least one complete CRISPR system, while seven strains lacked one [44]. Additionally, CRISPR genes were not identified in *C. butyricum* YF1. Pei et al. (2023) reported that only 28% (22 of 78) *C. butyricum* strains had Type IB CRISPR-Cas systems with 38 to 190 spacers, and prophage integration may be antagonized by the CRISPR system [62].

To further investigate the functional profile of the *C. butyricum* YF1 from a whole-genome perspective, functional annotation was conducted using the COG, GO, KEGG, KOG, NR, Pfam, Swiss-Prot, and TrEMBL databases. A total of 3146 genes, representing 99.1%, were successfully annotated. The annotation results of metabolic process, catalytic activity, and membrane part elucidated a strong correlation after comparison with the use of the GO database. The results of annotation with the COG database displayed that the highest number of genes were annotated to translation, ribosomal structure, and biogenesis, followed by carbohydrate transport and metabolism. In addition, the KEGG analysis provided a research platform to identify the secondary metabolic pathway of YF1, which exhibited that the highest number of genes were annotated to carbohydrate metabolism. Interestingly, these functional annotation results suggest that YF1 may possess exceptional metabolic pathways. This finding is similar to that of the *C. butyricum* LV1 strain, which had the highest number of genes annotated to metabolic pathways [26]. Moreover, glycolytic metabolic pathways and acetyl-CoA metabolic pathways used to produce butyric acid and acetic acid are complete in YF1. This could provide valuable insights for improving their production efficiencies. Additionally, genes classified as glycoside hydrolases (GHs) were found to be the most abundant in YF1 according to CAZy annotation, which aligns with the findings of *C. butyricum* LV1 strain [26]. Glycoside hydrolases can inhibit the adhesion and invasion of potentially pathogenic bacteria and their toxins in the epithelial cells of the intestinal mucosa [63]. Consequently, YF1 may offer protection against the colonization of pathogenic bacteria and help maintain the normal physiological functions of the intestine.

Small operons that encode toxins and antitoxins are essential for bacterial survival under dynamically changing environmental conditions [64]. Therefore, the genes producing virulence factors in the *C. butyricum* YF1 were predicted against the PHI and VFDB databases. In this study, among the 108 virulence-related genes co-predicted in PHI and VFDB, the mutant phenotype of YF1 was dominated by reduced virulence. This finding is consistent with the results of previous studies in *C. butyricum* LV1 [26]. The toxin genes were conserved in *C. butyricum*, including groEL (which is involved in the folding of newly synthesized proteins), clpP (which plays a role in various cellular processes and virulence in bacteria), and fbpA (a fibronectin-binding protein found on the listerial surface that mediates adherence to host cells and acts as a chaperone for two virulence factors, LLO and InlB) [44]. These conserved toxin genes were also present in YF1. In addition, the resistance genes in YF1 were predicted by the bacterial resistance databases CARD and ARDB. Yang et al. (2024) found that a resistance gene to fluoroquinolones was present in all 153 strains of *C. butyricum* [44]. This study identified the presence of 25 fluoroquinolone antibiotic genes in YF1. Additionally, 66 resistance genes on the circular chromosome which are found in both CARD and ARDB. These genes confer resistance to macrolides, vancomycin, tetracycline, teicoplanin, lincosamide, streptogramin, chloramphenicol, β-lactams, bacitracin, sulfonamides, and penicillin. Notably, the presence of the gene that confers resistance to neomycin was predicted on the circular plasmid, which corresponds to the antibiotic resistance to neomycin of YF1 in Table 1, confirming the resistance of YF1 to neomycin antibiotics.

## 5. Conclusions

In this study, a strain of *C. butyricum* was isolated from the intestine of *M. albus*, which exhibited a high growth rate and demonstrated the ability to produce high yields of acetic acid and butyric acid. Importantly, this strain also exhibited tolerance to high temperature, bile salts, and artificial intestinal and gastric fluids, while showing susceptibility to most antibiotics. Additionally, whole-genome data analysis revealed the functional profile, the butyric acid metabolism pathway, and profiling of resistance genes and virulence factors. Overall, our findings provide a theoretical basis for further research and the application of *C. butyricum* YF1.

## Figures and Tables

**Table 1:** Physiological and biochemical characteristics of *C. butyricum* YF1.

Test Items	Result	Test Items	Result
Acid production	+	MR	+
Gas production	+	V-P	+
Lactose	+	Citrate	−
Maltose	+	Hydrogen sulfide	−
Mannitol	+	Urea	−
Sugar	+	Semi-solid agar	+
Peptone water	−	Starch hydrolysis	+

**Table 2:** Analysis of antibiotic resistance of *C. butyricum* YF1.

Antibiotics	Content	Diameter of Inhibition Circle (mm)	Sensitivity
Penicillin	10 μg/tablet	31.23 ± 1.76	S
Ampicillin	10 μg/tablet	31.83 ± 0.34	S
Carbenicillin	100 μg/tablet	31.9 ± 0.43	S
Peracillin	100 μg/tablet	33.07 ± 0.48	S
Cephalexin	30 μg/tablet	32.1 ± 1.31	S
Cefazolin	30 μg/tablet	38.43 ± 1.14	S
Cefradine	30 μg/tablet	29.1 ± 0.42	S
Cefuroxime	30 μg/tablet	23.17 ± 0.6	S
Ceftriaxone	30 μg/tablet	34.23 ± 1.08	S
Cefoperazone	75 μg/tablet	38.4 ± 0.65	S
Tetracycline	30 μg/tablet	31.5 ± 0.51	S
Doxycycline	30 μg/tablet	30.07 ± 0.25	S
Minocycline	30 μg/tablet	28.53 ± 0.83	S
Erythromycin	15 μg/tablet	26.63 ± 0.71	S
Norfloxacin	10 μg/tablet	20.33 ± 1.45	S
Ofloxacin	5 μg/tablet	27.23 ± 0.62	S
Ciprofloxacin	5 μg/tablet	26.27 ± 0.12	S
Vancomycin	30 μg/tablet	26.47 ± 0.39	S
Furazolidone	300 μg/tablet	31.93 ± 0.33	S
Chloramphenicol	30 μg/tablet	36.4 ± 2.16	S
Clindamycin	2 μg/tablet	24.3 ± 1.42	S
Oxacillin	1 μg/tablet	10.83 ± 0.37	I
Amikacin	30 μg/tablet	13.07 ± 0.25	I
Kanamycin	30 μg/tablet	10.07 ± 0.09	I
Medemycin	30 μg/tablet	17.00 ± 1.20	I
Ceftazidime	30 μg/tablet	0.00 ± 0.00	R
Gentamicin	10 μg/tablet	0.00 ± 0.00	R
Neomycin	30 μg/tablet	0.00 ± 0.00	R
Polymyxin B	300 IU/tablet	0.00 ± 0.00	R
Cotrimoxazole	19 μg/tablet	0.00 ± 0.00	R

## Data Availability

The 16S rDNA sequence of *C. butyricum* strain YF1 from this article can be found in the GenBank data libraries under accession number PQ524489. The genome data of *C. butyricum* YF1 from this article can be found in the NCBI database under BioProject accession number PRJNA1178835.
